# Challenges in public healthcare research data warehouse integration and operationalisation.

**DOI:** 10.23889/ijpds.v7i3.1859

**Published:** 2022-08-25

**Authors:** Tanya Ravipati, Nadine E Andrew, Velandai Srikanth, Richard Beare

**Affiliations:** 1 NCHA

## Objectives

Public health service organisations use multiple patient administration and electronic health record systems. We describe the implementation of a data warehouse automation tool within the National Centre for Healthy Ageing (NCHA) data platform to operationalise a research data warehouse to optimise data quality and data provision for health services research.

## Approach

The traditional data warehouse life cycle comprises repetitive manual tasks and dependency on specialist developers. Automation tools overcome most of these inefficiencies. We conducted an internal risk benefit analysis which was validated by published literature containing data warehouse optimisation and automation. Industry-based data warehouse automation tools were reviewed to align the NCHA requirements with the tool’s functionality. Tools were then shortlisted and evaluated over a six-week period: (1) automation of standard tasks; (2) data pipeline alignment with the World Health Organization’s (WHO) Data Quality Review Framework; and (3) resource dependency risk mitigation through a Proof of Concept (PoC).

## Results

The priority areas identified by the risk benefit analysis included: end-to-end data warehouse automation; auto scripting; connectivity/linkage with multiple sources, reverse/forward engineering, audit trail conformance, scalability, multiple data warehouse architectures support, automated documentation; data management including data quality; and post-subscription independence. Twenty scientific publications were included in the final literature review (10% within healthcare) and supported the majority of identified priority areas. The industry-based review identified 11 suitable data warehouse/Extract-Transform-Load (ETL) automation tools. Five tools demonstrated adequate performance for task automation, data quality management, reduced dependency on specialist developers and on-premise linkage compatibility. Two automation tools were tested each for 6 weeks through PoC development. One automation tool met 8 out of the 10 automation requirements and was selected for implementation.

## Conclusion and Relevance

Data warehouse development processes are complex and time consuming. Tools that offer automation of repetitive tasks and scripting increase the consistency while reducing the dependency on specialist staff. Integrated data quality management minimises the time researchers spend in pre-processing patient level data sourced through a semi-automated data warehouse.

